# GR Utilizes a Co-Chaperone Cytoplasmic CAR Retention Protein to Form an N/C Interaction

**DOI:** 10.1177/1550762918801072

**Published:** 2018-10-24

**Authors:** Marumi Ohno, Masahiko Negishi

**Affiliations:** 1 National Institute of Environmental Health Sciences, Research Triangle Park, NC, USA

**Keywords:** CCRP, GR, N/C interaction

## Abstract

The N-terminal domain (NTD) of nuclear receptor superfamily members has been recently reported to regulate functions of the receptor through the interaction between the NTD and the C-terminal ligand binding domain (LBD), so-called an N/C interaction. Although this N/C interaction has been demonstrated in various nuclear receptors, eg, androgen receptor, this concept has not been observed in glucocorticoid receptor (GR). We hypothesized that GR requires its co-chaperone CCRP (cytoplasmic constitutive active/androstane receptor retention protein) to form a stable N/C interaction. This hypothesis was examined by co-immunoprecipitation assays using GR fragments overexpressing COS-1 cell lysate. Here, we demonstrated that GR undergoes the N/C interaction between the ^26^VMDFY^30^ motif in the NTD and the LBD. More importantly, co-chaperone CCRP is now found to induce this interaction. By the fact that a negative charge at Y30 disrupts this interaction, this residue, a potential phosphorylation site, was indicated to regulate the GR N/C interaction critically. Utilizing Y30F and Y30E mutants as N/C interacting and noninteracting forms of GR, respectively, a 2-dimensional blue native/sodium dodecyl sulfate-polyacrylamide gel electrophoresis was performed to examine whether or not the N/C interaction regulated formation of GR complexes. A cDNA microarray analysis was performed with COS-1 cells expressing Y30F or Y30E. We will present experimental data to demonstrate that CCRP is essential for GR to form the N/C interaction and will discuss its implications in GR functions.

## Abbreviations

ANOVA, analysis of variance; AR, androgen receptor; CAR, constitutive active/androstane receptor; CBB, Coomassie brilliant blue; CCRP, cytoplasmic constitutive active/androstane receptor retention protein; DBD, DNA binding domain; ER, estrogen receptor; EYFP, enhanced yellow fluorescent protein; GFP, green fluorescent protein; GR, glucocorticoid receptor; HRP, horseradish peroxidase; HSP, heat shock protein; IP, immunoprecipitation; LBD, ligand binding domain; MR, mineralocorticoid receptor; NTD, N-terminus domain; PR, progesterone receptor; TPR, tetratricopeptide repeat; 2D-BN/SDS-PAGE, two-dimensional blue native/sodium dodecyl sulfate-polyacrylamide gel electrophoresis.

## Introduction

Nuclear receptors are, in general, defined as ligand-activated transcription factors. They are featured by their domain structures with N-terminus domain (NTD), DNA binding domain (DBD), and C-terminal ligand binding domain (LBD). Transcriptional activation functions are present in the NTD and the LBD of nuclear receptors. The LBD regulates ligand-dependent transcriptional activities whereas the functions of NTD are thought to be constitutively activated and to be somehow suppressed by the LBD. Recent studies have been increasingly emphasized an interdomain interaction between the NTD and the LBD, so-called an N/C interaction, as a regulatory determinant of nuclear receptor functions. Androgen receptor (AR) is the best-known nuclear receptor that undergoes an N/C interaction to fully elicit its transcriptional activity. The interaction is mediated by the binding of the LBD to a specific FXXLF sequence in the NTD and serves to modulate protein-protein interactions of AR.^1-4^ The N/C interaction regulates AR functions including protein stability, dimerization, and gene activation.^5-8^ The N/C interaction-defective mouse model (deletion of FXXLF) showed a clear delay of neurodegeneration induced by aggregation of AR with an expanded glutamine repeat, further confirming a physiological importance of this interdomain interactions for nuclear receptor regulation.^5^ Whereas the N/C interactions were also demonstrated in various other nuclear receptors, progesterone receptor (PR), estrogen receptor alpha (ERα), and mineralocorticoid receptor (MR), this concept has not yet been observed in glucocorticoid receptor (GR).^9-11^ Whereas the N/C interactions of AR, PR, and MR are induced by ligand binding, the ERα N/C interaction does not require ligands and is suggested to be modified by cell-specific factors such as co-chaperone proteins.^3,9-11^ It brought us a hypothesis that GR utilizes a co-chaperone protein to form a stable N/C interaction. Here, we have investigated whether GR requires co-chaperone CCRP (cytoplasmic constitutive active/androstane receptor retention protein) for the N/C interaction and what the biological significance is.

CCRP is a member of heat shock protein (HSP) 40/DNAJ family with a characteristic J-domain. Having 2 tetratricopeptide (TPR) motifs, CCRP is also characterized as a protein that belongs to the TPR family. Both J- and TPR-domains mediate protein-protein interactions, suggesting that CCRP modulates intermolecular and intramolecular interactions. Our group previously demonstrated an interaction of CCRP with nuclear receptor constitutive active/androstane receptor (CAR) to stabilize a CAR-HSP90 complex and retain the receptor in the cytoplasm of HepG2 cells, with which the name cytoplasmic CAR retention protein (CCRP) was coined to this protein.^12^ Subsequently, CCRP knockout (KO) mice were utilized to determine the role of CCRP in CAR-mediated activation of *Cyp2b10* gene in the liver.^13^ In addition to CAR, CCRP has been shown to interact with other nuclear receptors including pregnane X receptor, PR, AR, MR, ERα, and GR.^14-17^ However, a role of CCRP in the regulation of nuclear receptor functions is largely unclear.

In the present study, co-immunoprecipitation (co-IP) assays were employed to determine an N/C interaction of GR between a short peptide (^26^VMDFY^30^) near the N-terminus of NTD and the LBD in COS-1 cells. Then, these co-immunoprecipitations were performed with or without co-expression of CCRP to confirm the regulatory roles of CCRP in this N/C interaction. In addition, utilizing the fact that phosphor-mimic mutation of tyrosine within the VMDFY motif to glutamic acid abolished this N/C interaction, either GR Y30F or GR Y30E mutant was ectopically expressed in COS-1 cells for a subsequent 2-dimensional blue native/sodium dodecyl sulfate-polyacrylamide gel electrophoresis (2D-BN/SDS-PAGE). Here, we have presented experimental evidence that GR undergoes the CCRP-mediated N/C interaction, and a possibility that GR regulates different functions through the N/C interaction is discussed.

## Materials and Methods

### Plasmid Construction

The plasmids used in this study included FLAG-6c-CMV-hGRα (referred as FLAG-GR; full length, 1-777; ΔLBD, 1-527; ΔNTD, 1-25/394-777; LBD, 528-777; Δ26-76, 1-25/77-777; Y30F and Y30E), enhanced yellow fluorescent protein (EYFP)-c1-hGRα-26-76 (referred as EYFP-26/76; WT, AADFY, VMDAA, Y30F, and Y30E), and pcDNA3.1-mCCRP-V5 (referred as CCRP-V5). EYFP-c1-hGRα was a kind gift from Dr Cidlowski (National Institute of Environmental Health Sciences, Research Triangle Park, North Carolina). GRα coding region was subcloned into a FLAG-6c-CMV vector. Mutations were introduced using a Prime STAR MAX DNA polymerase (Takara, Otsu, Japan) according to the manufacturer’s instruction. The CCRP-V5 expression vector was obtained as previously described.^12^ Sequences of all plasmids were confirmed by DNA sequencing.

### Cell Culture and Transfection

The African green monkey kidney cell line, COS-1 cells were cultured in Dulbecco’s Modified Eagle Medium (11965-092, Invitrogen, Carlsbad, California) supplemented with 10% fetal bovine serum in a humidified 5% CO_2_ incubator at 37°C. Endogenous expression of CCRP in COS-1 cells was examined by Western blot analysis and found to be not detected (Supplemental Figure 1). COS-1 cells were transiently transfected with expression plasmids by reverse transfection technique using FuGENE 6 (Promega, Madison, Wisconsin) according to the manufacturer’s protocol. After 40 hours post-transfection, cells were used for each experiment as described individually below.

### Co-IP Assay

Schematic representation of FLAG- or EYFP-tagged GR fragments is shown in Figure 2b. COS-1 cells were lysed in cold IP buffer (20 mM Tris-HCl, pH 7.5, 150 mM NaCl, 1 mM EDTA, 1% Triton-X) containing protease inhibitor cocktail (Roche Diagnosis, Mannheim, Germany) and sonicated briefly to obtain whole lysates. Co-IP was carried out using FLAG-agarose affinity gel (A2220, Sigma, St. Louis, Missouri) or anti-V5 antibody (46-0705, Invitrogen) combined with Dynabeads protein G (Invitrogen) for 2 to 4 hours at 4°C. After the incubation, resin or beads were washed in cold tris-buffered saline (TBS) (for FLAG-agarose resin) or IP buffer (for Dynabeads) 4 times. Immunoprecipitated proteins were eluted in 2× SDS sample buffer by heating at 70°C for 10 min. Eluted proteins were subjected to Western blot analysis.

### Western Blot Analysis

Proteins were separated with 8.5% SDS-PAGE and transferred to polyvinylidene difluoride (PVDF) membrane. After blocking with 5% nonfat dry milk containing TBS-0.1% Tween 20 buffer, membrane was probed with antibodies in the blocking buffer for overnight at 4°C. For detection of FLAG-GR, CCRP-V5, and EYFP-26/76, horseradish peroxidase (HRP) conjugating anti-FLAG antibody (1:5000, A8592, Sigma), anti-V5 antibody (1:5000, 46-0708, Invitrogen), and anti-green fluorescent protein (GFP) antibody (1:10000, ab6663, Abcam, Cambridge, UK) were used, respectively. Protein bands on membrane were visualized using enhanced chemiluminescence detection reagent (Advansta, Menlo Park, California).

### Two-Dimensional Blue Native/Sodium Dodecyl Sulfate-Polyacrylamide Gel Electrophoresis

Two-Dimensional Blue Native/Sodium Dodecyl Sulfate-Polyacrylamide Gel Electrophoresis (2D-BN/SDS-PAGE) is a powerful tool to analyze multiprotein complexes. Combined BN-PAGE with SDS-PAGE was shown to result in the separation of several individual subunits of the resolved complexes, offering an interesting 2-dimensional electrophoresis approach that allows us to profile target molecule containing protein complexes. According to Wittig et al,^18^ 2D-BN/SDS-PAGE was performed with modifications as follows. Cells were lysed in low salt HEGMS buffer (20 mM HEPES pH 7.6, 50 mM NaCl, 15% Glycerol, 1 mM Na_2_MoO_4_, 1 mM EDTA) containing protease inhibitor cocktail (Roche) and sonicated briefly. For a Triton-X containing experiment, low salt HEGMS buffer containing 0.5% Triton-X was used for sample preparation. After the centrifugation at 11 000 g for 5 minutes, clear supernatants were collected as whole lysates. Then, one-tenth volume of 10× BN sample buffer (2.5% CBB G-250, 100 mM Bis-Tris-HCl, pH 7.0, 500 mM 6-aminocaproic acid) was added to each sample. Samples were incubated on ice for 5 to 10 minutes, and 20 µg proteins were loaded to a 4% to 16% gradient native gel (Invitrogen). The first dimensional electrophoresis was performed using appropriate cathode buffer (50 mM Tricine-HCl, 15 mM Bis-Tris-HCl, pH 7.0, 0.02% CBB G-250) and anode buffer (50 mM Bis-Tris-HCl, pH 7.0) at 4°C to 7°C for 2 to 3 hours. Power supply was set at 150 V. After the electrophoresis, each lane was excised from the first dimensional BN gel and incubated in SDS-PAGE running buffer for 15 to 20 minutes. Each excised gel was placed onto an 8.5% SDS denaturing polyacrylamide gel and covered with an SDS stacking gel. After the polymerization, the second dimensional electrophoresis was performed at 180 V for 1 hour at room temperature. Then, a Western blot analysis was carried out as described above.

### Nuclear and Cytoplasmic Protein Preparation

After 1 hour treatment with 0.1% dimethyl sulfoxide (DMSO) or 100 nM dexamethasone, nuclear and cytoplasmic proteins were extracted from COS-1 cells using NE-PER kit (PIERCE, Rockford, Illinois) according to the manufacturer’s protocol. Then, a Western blot analysis was carried out as described above. To confirm successful fractionation, protein levels of HSP90 and histone deacetylase 1 (HDAC1) were determined with anti-HSP90 antibody (1:1000, 610419, BD Transduction Laboratories, San Jose, California) and anti-HDAC1 antibody (1:1000, 2062, Cell Signaling Technology, Danvers, Massachusetts), respectively.

## Results

### CCRP Facilitated the N/C Interaction Within GR

**Figure 1.**
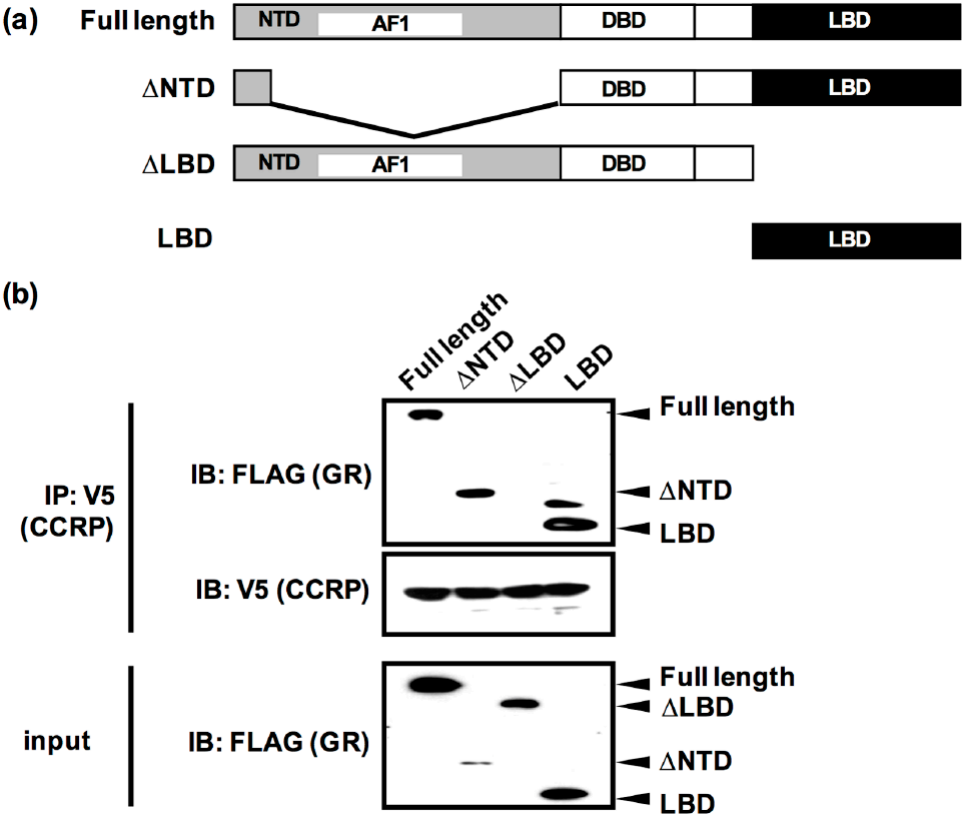
CCRP interacted with the GR LBD. *Note.* (a) Schematic representation of FLAG-tagged GR proteins (full length, ΔNTD, ΔLBD, and LBD) used in co-IP assays. (b) Interaction between CCRP and GR. CCRP-V5 and intact or truncated FLAG-GR were transiently expressed in COS-1 cells. Whole lysates were prepared and co-immunoprecipitation was performed using anti-V5 antibody. Co-immunoprecipitated proteins were eluted and subjected to Western blot analysis. Anti-FLAG antibody and anti-V5 antibody were used to detect FLAG-GR and CCRP-V5, respectively. CCRP = cytoplasmic constitutive active/androstane receptor retention protein; GR = glucocorticoid receptor; LBD = ligand binding domain; NTD = N-terminus domain.

To confirm a previous finding that CCRP binds to LBD of GR,^17^ a co-IP analysis was conducted using COS-1 cells overexpressing GR deletion mutants (Figure 1a). As expected, GR∆LBD did not interact with CCRP while full length GR and GR LBD were co-immunoprecipitated with CCRP (Figure 1b). This result confirmed the previous finding that LBD is sufficient for the interaction with CCRP.

**Figure 2.**
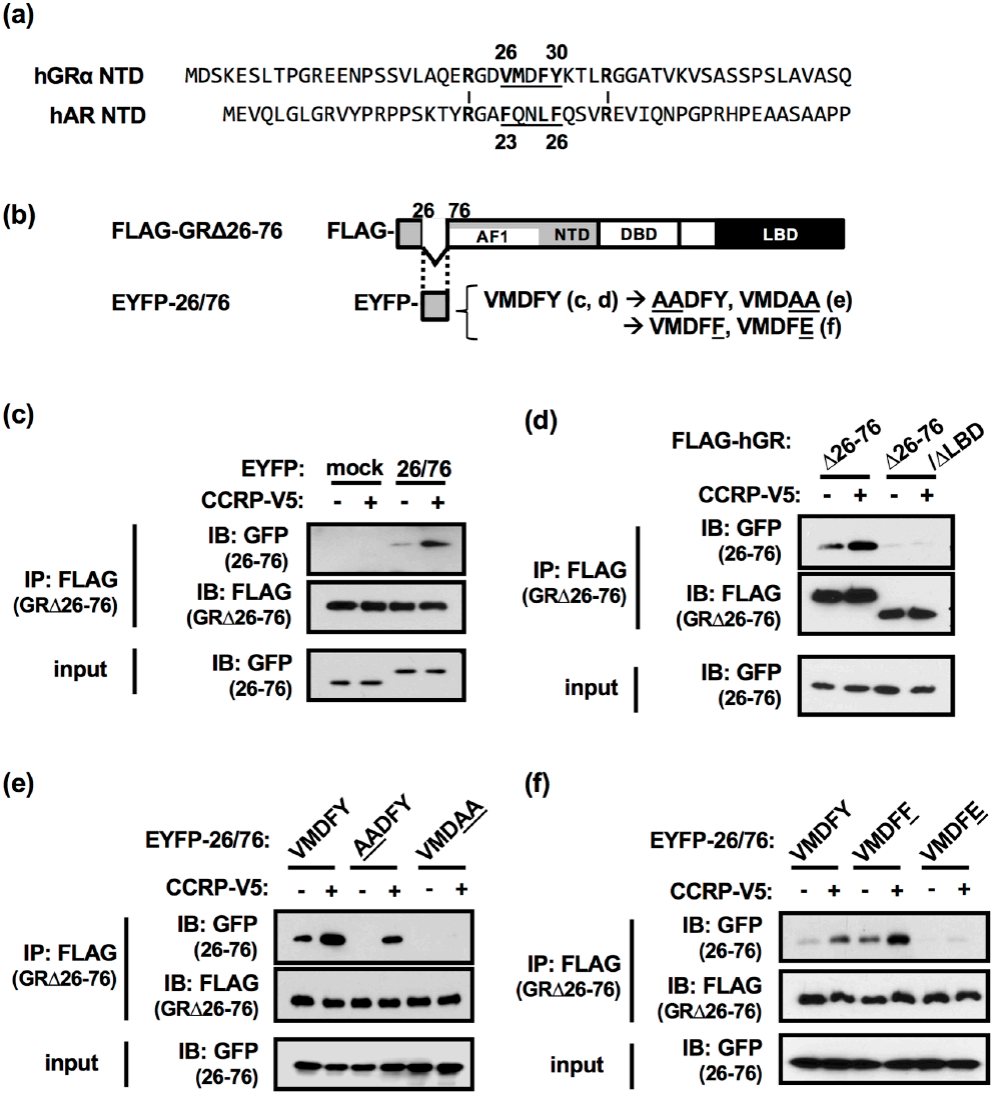
CCRP facilitated the GR N/C interaction between the VMDFY residues and the LBD. *Note.* (a) Partial amino acid sequences of the NTD regions of human GR and AR. The AR FQNLF and the GR VMDFY residues are underlined, and hydrophobic residues within these residues are shown in bold. Arginine residues which have additive effects on the AR N/C interaction and corresponding arginine residues in the GR NTD are also shown in bold. Flanking numbers above and below the sequences indicate amino acid residue position. (b) Schematic representation of FLAG- and EYFP-tagged GR fragments used in co-IP assays. Mutations with in VMDFY motif are also indicated. (c) Interaction of FLAG-GRΔ26-76 with EYFP-mock or EYFP-26/76 in the absence or presence of CCRP-V5. (d) Interaction of FLAG-GRΔ26-76 or FLAG-GRΔ26-76ΔLBD with EYFP-26/76 in the absence or presence of CCRP-V5. (e) Interaction of FLAG-GRΔ26-76 with EYFP-26/76 (WT, VMDFY; AADFY; VMDAA) in the absence or presence of CCRP-V5. (f) Interaction of FLAG-GRΔ26-76 with EYFP-26/76 (WT, VMDFY; Y30F, VMDFF; Y30E, VMDFE) in the absence or presence of CCRP-V5. (c-f) EYFP-26/76, FLAG-GRΔ26-76, and CCRP-V5 or pcDNA3.1-V5 were transiently expressed. Whole lysates were prepared in IP buffer and co- immunoprecipitation assays were performed using anti-FLAG agarose. Co-immunoprecipitated proteins were eluted and subjected to Western blot analysis. Anti-FLAG antibody and anti-GFP antibody were used to detect FLAG-GRΔ26-76 and EYFP-26/76, respectively. CCRP = cytoplasmic constitutive active/androstane receptor retention protein; GR = glucocorticoid receptor; LBD = ligand binding domain; NTD, N-terminus domain; AR = androgen receptor; EYFP = enhanced yellow fluorescent protein; GFP = green fluorescent protein.

Figure 2a shows partial amino acid sequences of GR and AR NTD regions. AR has ^23^FXXLF^26^ motif in the NTD which forms an α-helix and interacts with LBD.^1-4^ In addition to this core motif, 2 arginine residues flanking the motif have additive effects on the AR N/C interaction.^19^ When focusing on the hydrophobicity of amino acids, a similarity in the NTD was found between AR and GR. At the corresponding positions to those in the AR NTD, the GR NTD has hydrophobic amino acid residues, V, F, and Y within the ^26^VMDFY^30^ sequence, and 2 arginine residues near the peptide. The ^26^VMDFYKT^32^ peptide was predicted to form an α-helical conformation by GOR IV, a secondary structure prediction tool.^20^ Thus, in terms of N/C interactions, the GR VMDFY residues were expected to be equivalent in function to the FXXLF motif from the AR NTD.

To examine the interaction between the VMDFY containing region and the LBD and a role of CCRP for the interaction, a series of co-IP assays was performed. FLAG-tagged GR with deletion of 26-76 residues (referred as FLAG-GR∆26-76) and EYFP-tagged 26-76 residues (referred as EYFP-26/76) were ectopically expressed in COS-1 cells in the presence or absence of CCRP-V5 (Figure 2b). Utilizing total lysates, whether or not EYFP-26/76 was co-immunoprecipitated with FLAG-GR∆26-76 was checked by co-IP assays with FLAG affinity resin. For the detection of EYFP-26/76, anti-GFP antibody was used. As expected, EYFP-26/76 was co-immunoprecipitated with FLAG-GR∆26-76 while EYFP alone did not interact with FLAG-GR∆26-76 at all (Figure 2c, lane 1 vs 3). Moreover, the interaction between EYFP-26/76 and FLAG-GR∆26-76 was increased by the co-expression of CCRP-V5 (Figure 2c, lane 3 vs 4). This interaction was almost completely abolished by the deletion of LBD (Figure 2d, lane 1, 2 vs 3, 4). In addition, only LBD tagged with FLAG was able to bind to EYFP-26/76 in the presence of CCRP-V5 (data not shown). But, the interaction was more stable in the combination with FLAG-GR∆26-76 for unknown reason. That is why FLAG-GR∆26-76 instead of FLAG-LBD was used in this study.

Next, the contribution of hydrophobic amino acid residues of the VMDFY sequence to the interaction was investigated by mutation assays. Amino acid substitution from VMDFY to VMD*AA* completely abolished the interaction (Figure 2e, lane 1, 2 vs 5, 6) while VMDFY to *AA*DFY substitution had less effect (Figure 2e, lane 1, 2 vs 3, 4). When tyrosine (Y30) was substituted to a phosphor-mimicking glutamic acid (VMDF*E*) to introduce a negative charge, the interaction was greatly decreased (Figure 2f, lane 1, 2 vs 5, 6) although the interaction of FLAG-GR∆26-76 with EYFP-26/76 was increased by amino acid substitution to non-phosphor-mimicking phenylalanine (VMDF*F*) (Figure 2f, lane 1, 2 vs 3, 4). These results demonstrated that GR forms an N/C interaction between the VMDFY motif and the LBD in a Y30-dependent manner and that CCRP facilitates the GR N/C interaction.

**Figure 3.**
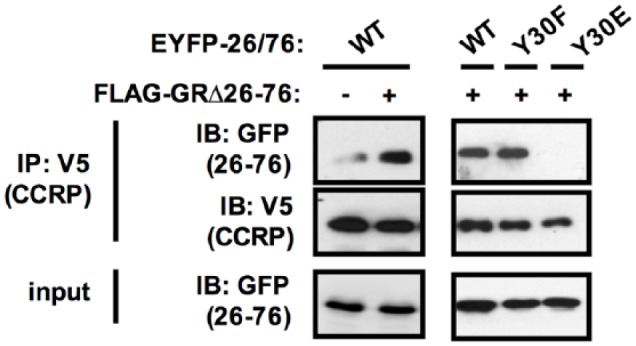
CCRP promoted the N/C interaction. *Note.* Interaction of CCRP-V5 with EYFP-26/76 (WT, VMDFY; Y30F, VMDFF; Y30E, VMDFE) in the absence or presence of FLAG-GRΔ26-76. CCRP-V5, EYFP-26/76, and FLAG-GRΔ26-76 or pcDNA3.1-V5 were transiently expressed. Whole lysates were prepared and co-immunoprecipitation was performed using anti-V5 antibody. Co-immunoprecipitated proteins were eluted and subjected to Western blot analysis. Anti-V5 antibody and anti-GFP antibody were used to detect CCRP-V5 and EYFP-26/76, respectively. CCRP = cytoplasmic constitutive active/androstane receptor retention protein; EYFP = enhanced yellow fluorescent protein; GR = glucocorticoid receptor; GFP = green fluorescent protein.

Furthermore, as shown in Figure 3, the interaction between CCRP-V5 and EYFP-26/76 was also greatly increased by co-expression of FLAG-GR∆26-76 although EYFP-26/76 bearing Y30E mutation did not interact with CCRP-V5 even in the presence of FLAG-GR∆26-76. Taken together with results shown in Figure 2, it was indicated that CCRP interacts with the GR LBD and recruits the VMDFY motif in the NTD to form the N/C interaction.

**Figure 4.**
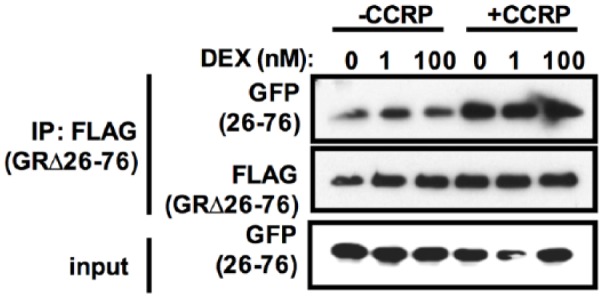
DEX had no effect on the N/C interaction. *Note.* FLAG-GRΔ26-76, EYFP-26/76, and CCRP-V5 or pcDNA3.1-V5 were transiently expressed and cells were treated with 0, 1, or 100 nM DEX for 1 hour at 37°C in 5% CO_2_ incubator. Whole lysates were prepared in IP buffer and co-immunoprecipitation was performed using anti-FLAG agarose. Co-immunoprecipitated proteins were eluted and subjected to Western blot analysis. Anti-FLAG antibody and anti-GFP antibody were used to detect FLAG-GRΔ26-76 and EYFP-26/76, respectively. GR = glucocorticoid receptor; EYFP = enhanced yellow fluorescent protein; CCRP = cytoplasmic constitutive active/androstane receptor retention protein; DEX = dexamethasone; GFP = green fluorescent protein.

The effect of dexamethasone, a potent GR ligand, on the N/C interaction was also investigated. Treatment of COS-1 cells with 1 and 100 nM dexamethasone had no effect on the interaction between EYFP-26/76 and FLAG-GR∆26-76 regardless of CCRP-V5 expression (Figure 4). This result suggested that the GR N/C interaction is ligand-independent at least under our experimental conditions.

### The CCRP-Mediated N/C Interaction Critically Regulated Protein-Protein Interactions of GR

**Figure 5.**
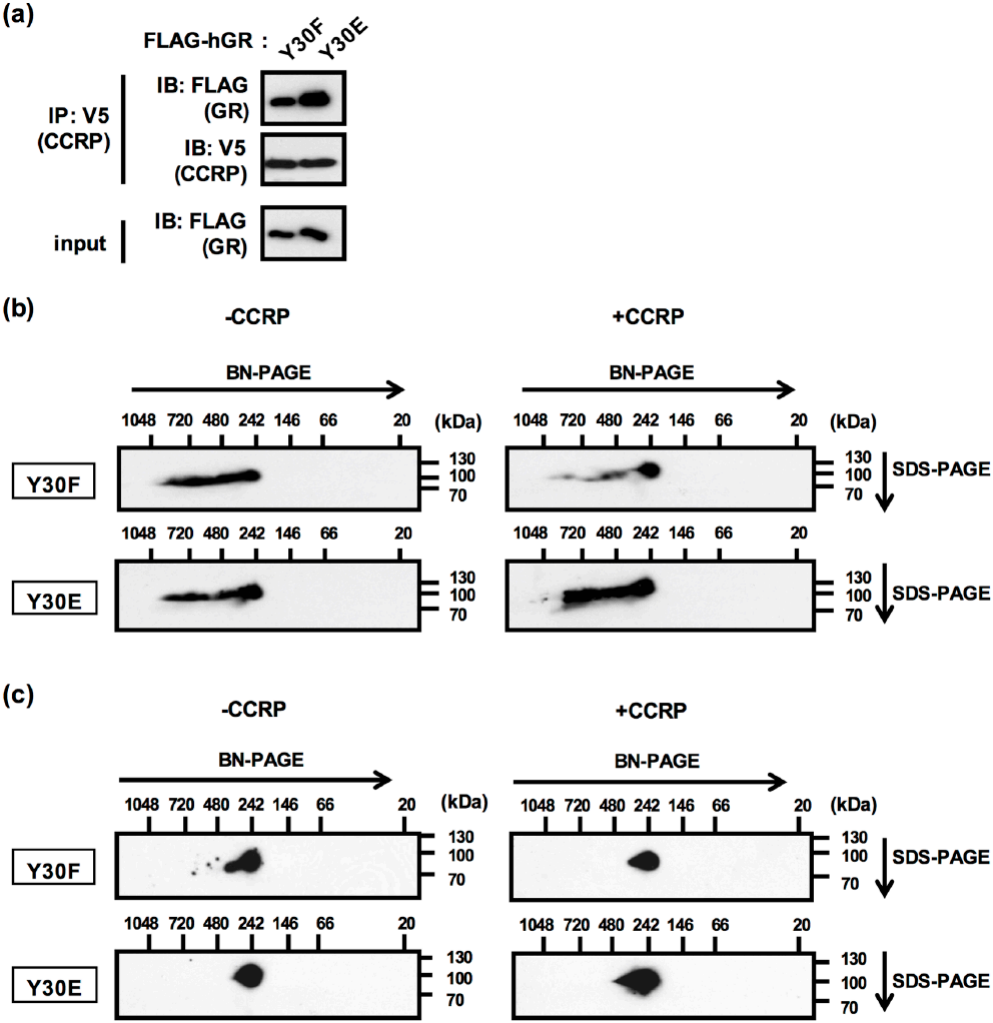
The CCRP-mediated N/C interaction determined GR protein complexes. *Note.* (a) Interaction between CCRP-V5 and FLAG-GR bearing Y30F or Y30E mutation. CCRP-V5 and FLAG-GR (Y30F or Y30E) were transiently expressed. Whole lysates were prepared and co-immunoprecipitation was performed using anti-V5 antibody. Co-immunoprecipitated proteins were eluted and subjected to Western blot analysis. Anti-V5 antibody and anti-FLAG antibodies were used to detect CCRP-V5 and FLAG-GR, respectively. (b, c) 2D-BN/SDS-PAGE analyses showed that the CCRP-mediated N/C interaction dissociated proteins from GR protein complex detected at around 242 kDa. FLAG-GR (Y30F or Y30E) and CCRP-V5 or pcDNA3.1-V5 were transiently expressed and total lysates were prepared in low salt HEGMS buffer (b) or 0.1% Triton-X containing low salt HEGMS buffer (c). Samples were loaded on a 4% to 16% BN gel (the first dimension) followed by the second dimension separation using 8.5% SDS-PAGE gels and Western blotting. Anti-FLAG antibody detected FLAG-GR at around 100 kDa as expected. CCRP = cytoplasmic constitutive active/androstane receptor retention protein; GR = glucocorticoid receptor; 2D-BN/SDS-PAGE = 2-dimensional blue native/sodium dodecyl sulfate-polyacrylamide gel electrophoresis.

The effect of the CCRP-mediated N/C interaction was further investigated with 2D-BN-SDS-PAGE technique, which allows us to know total molecular weight of multiple protein complexes. GR Y30F and Y30E mutants were utilized as N/C interacting and noninteracting models. First, interactions of CCRP with Y30F and Y30E mutants were confirmed by co-IP assay (Figure 5a). Both Y30F and Y30E bearing GR were detected from 242 to over 1000 kDa in the absence of CCRP (Figure 5b, left panels). Surprisingly, most of GR Y30F co-expressed with CCRP was detected at 242 kDa while CCRP did not change the distribution pattern of GR Y30E (Figure 5b, right panels). Thus, co-expression of CCRP decreased high molecular weight protein complexes only when the GR N/C interaction could occur. Moreover, the addition of Triton-X dissociated protein interactions at high molecular weight, and only 242-kDa complex was remained in all samples (Figure 5c). These data indicated that the 242-kDa complex is formed independently of CCRP or the N/C interaction, suggesting that CCRP-mediated N/C interaction inhibits protein interactions with the 242-kDa GR complex.

### The N/C Interaction Did Not Regulate Intracellular Localization of GR

**Figure 6.**
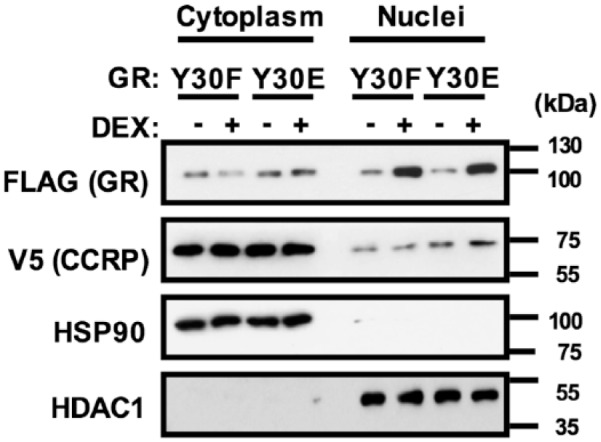
GR does not require the GR N/C interaction for nuclear translocation. *Note.* Western blot analysis showed intracellular localization of GR bearing Y30F or Y30E mutation after treatment with vehicle or dexamethasone. CCRP-V5 and FLAG-GR (Y30F or Y30E) were transiently expressed and cells were treated with vehicle (0.1% DMSO) or 100 nM dexamethasone for 1 hour at 37°C in a 5% CO_2_ incubator. Cytoplasmic and nuclear proteins were extracted, followed by Western blot analysis. Anti-FLAG and V5 antibodies were used to detect FLAG-GR and CCRP-V5, respectively. Fractionation was confirmed by abundance of control proteins (HSP90 for cytoplasm and HDAC1 for nuclei). GR = glucocorticoid receptor; CCRP = cytoplasmic constitutive active/androstane receptor retention protein; DMSO = dimethyl sulfoxide.

CCRP-V5 and GR Y30F or Y30E were overexpressed in COS-1 cells, and cells were treated with vehicle DMSO or 100 nM dexamethasone for 1 hour. Their intracellular localizations were determined by Western blots (Figure 6). CCRP was mainly localized in the cytoplasm before and after DEX treatment. GR Y30E, which was unable to form the N/C interaction, accumulated in the nucleus after DEX treatment as observed with the Y30F. Thus, it was suggested that the N/C interaction plays no role in the regulation of GR nuclear in response to DEX.

## Discussion

Here, we have demonstrated that CCRP facilitates GR to form the N/C interaction. Although deletion of the LBD enabled GR to elicit the NTD-dependent transcriptional activity, attempts to demonstrate any types of intramolecular interaction to support this NTD function have not been successful. For example, the LBD was unable to repress the NTD-dependent transactivation of a reporter gene when C- and N-terminal GR fragments were co-expressed in cell-based transfection assays.^21^ Mammalian 2-hybrid assays also failed to show the LBD/NTD interaction.^11^ Noticeably, these previous experiments were performed in CV-1 or CV-1-derived COS-1 cells. These cells are not suitable to investigate the N/C interaction as they do not express endogenous CCRP and because CCRP is essential for GR to form the interaction. In supporting the role of CCRP in the interaction, yeast Hsp40 Ydj1, which interacts with GR via its J-domain enabled the GR LBD to repress its NTD-mediated transcription activity in yeast-based reporter assays.^22^

**Figure 7.**
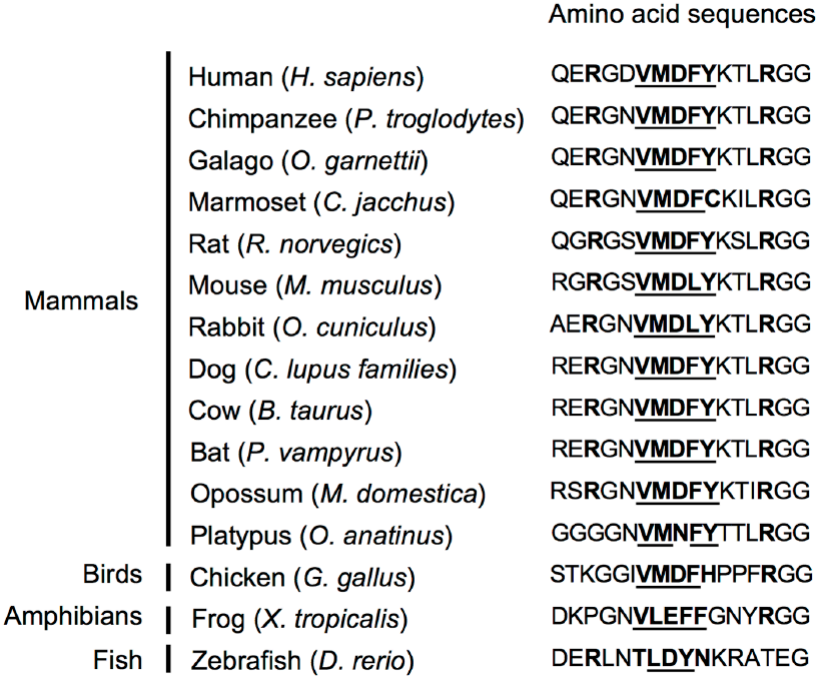
GR conserves the VMDFY motif cross species. *Note.* The VMDFY motifs and flanking arginine residues are shown in bold and residues of which polarity is similar to the VMDFY motif at each corresponding position are underlined. GR = glucocorticoid receptor.

The ^23^FQNLF^26^ peptide of AR, which regulates the N/C interaction, is generally represented as the FXXLF motif. It is because the hydrophobic residues at both ends determine functionality as indicated by the fact that either *A*QNLF or FQN*AA* mutants abolished the AR N/C interaction.^2^ In GR, whereas ^26^*AA*DFY^30^ mutant modestly decreased N/C interaction, ^26^VMD*AA*^30^ mutant abolished it. Thus, the functionality of the ^26^VMDFY^30^ peptide somewhat resembles that of the FXXLF motif in AR. However, what is unique to GR was that the motif peptide ends by tyrosine, of which single mutation to a similar size negatively charged glutamic acid (Y30E), but not to phenylalanine (Y30F), was sufficient to abolish the N/C interaction of GR. Thus, Y30 appears to be the most critical residue for GR to regulate the N/C interaction. As glutamic acid often exhibits a characteristic of phosphorylated tyrosine, the VMDFY motif could be phosphorylated to regulate the N/C interaction. As the phosphorylation of AR at S16 near to the FXXLF motif was shown to decrease the AR N/C interaction, the phosphorylation-dependent regulation of the N/C interaction of nuclear receptors has been suggested.^5^ Cell signals eliciting phosphorylation and the protein kinase that phosphorylates Y30 must be identified to further understanding of the GR N/C interaction in future investigations. In addition, the VMDFY motif in GR is highly conserved among species, especially mammals (Figure 7). It suggests an importance of the VMDFY motif in the regulation of GR functions.

Our 2D-BN/SDS-PAGE analysis showed that CCRP regulates GR protein complexes in an Y30-dependent manner. Both Y30E and Y30F formed a streak of larger complexes beginning with a size of approximately 242 kDa. Hedman et al reported the presence of multiple GR complexes with sizes ranging from 250 to 400 kDa in rat liver cytosolic fractions.^23^ Thus, GR complexes formed in COS-1 cells are similar to those observed in rat livers. CCRP dissociated all Y30F, but not Y30E, complexes greater than 242 kDa in size. Based on its size, this 242-kDa protein may be a complex with 2 GR monomers. In fact, GR was previously shown to form a homodimer ligand independently.^24^ From the result that the 242-kDa complex was formed regardless of CCRP or mutations at Y30, the N/C interaction may not affect the GR homodimerization. The present study also showed that the intracellular localization of GR in the absence or presence of dexamethasone was not affected by the N/C interaction. Instead, because regions of the NTD and the LBD provide interaction surfaces to transcription-related proteins, the GR N/C interaction would modify interactions with these proteins as reported for AR.^1-4^ The N/C interaction may enable GR to regulate unique genes as suggested by cDNA microarrays (Supplemental Figure 2).

**Figure 8.**
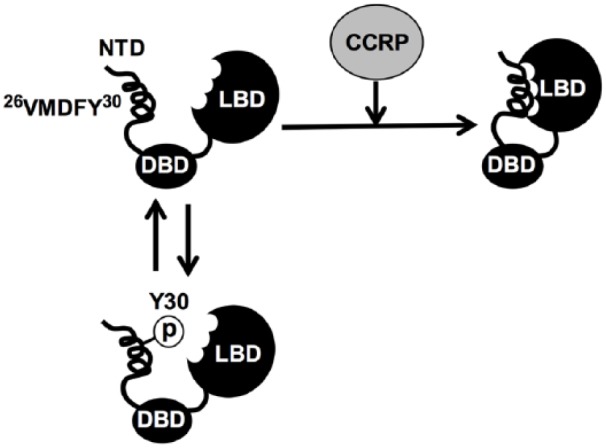
Schematic representation of the CCRP-mediated GR N/C interaction. *Note.* Based on experimental results, the role of CCRP in the N/C interaction is modeled. GR is unable to form the N/C interaction when Y30 is phosphorylated. Upon dephosphorylation, GR enables for CCRP to stimulate the interaction. CCRP appears to dissociate from GR after it formed the interaction since interaction of CCRP with GR deleted with NTD and Y30E, which do not form the N/C interaction, was more stable than that with full length and wild type GR. CCRP = cytoplasmic constitutive active/androstane receptor retention protein; GR = glucocorticoid receptor; NTD = N-terminus domain.

Collectively, we have now demonstrated that a GR peptide near the N-terminus (^26^VMDFY^30^) interacts with the LBD and forms the N/C interaction. Moreover, co-chaperone CCRP was found to bind the LBD to facilitate the GR N/C interaction. We show an expected model of the CCRP-mediated GR N/C interaction in Figure 8. While N/C interactions were previously observed in various nuclear receptors (eg, AR, PR, ER, and MR), GR is the first nuclear receptor for which the N/C interaction is regulated by a co-chaperone. Our current findings will provide new insights into how co-chaperone proteins regulate nuclear receptors and how nuclear receptors regulate different genes and different pathways.

## Supplemental Material

Ohno_Supplemental_Fig_1 – Supplemental material for GR Utilizes a Co-Chaperone Cytoplasmic CAR Retention Protein to Form an N/C Interaction Click here for additional data file. Supplemental material, Ohno_Supplemental_Fig_1 for GR Utilizes a Co-Chaperone Cytoplasmic CAR Retention Protein to Form an N/C Interaction by Marumi Ohno and Masahiko Negishi in Nuclear Receptor Signaling

## Supplemental Material

Ohno_Supplemental_Fig_2 – Supplemental material for GR Utilizes a Co-Chaperone Cytoplasmic CAR Retention Protein to Form an N/C Interaction Click here for additional data file. Supplemental material, Ohno_Supplemental_Fig_2 for GR Utilizes a Co-Chaperone Cytoplasmic CAR Retention Protein to Form an N/C Interaction by Marumi Ohno and Masahiko Negishi in Nuclear Receptor Signaling

## Supplemental Material

Supplemental_Figure_Captions – Supplemental material for GR Utilizes a Co-Chaperone Cytoplasmic CAR Retention Protein to Form an N/C Interaction Click here for additional data file. Supplemental material, Supplemental_Figure_Captions for GR Utilizes a Co-Chaperone Cytoplasmic CAR Retention Protein to Form an N/C Interaction by Marumi Ohno and Masahiko Negishi in Nuclear Receptor Signaling

## References

[1] He B Kemppainen JA Voegel JJ Gronemeyer H Wilson EM. Activation function 2 in the human androgen receptor ligand binding domain mediates interdomain communication with the NH(2)-terminal domain. J Biol Chem. 1999;274:37219-37225. 10601285 10.1074/jbc.274.52.37219

[2] He B Kemppainen JA Wilson EM. FXXLF and WXXLF sequences mediate the NH2-terminal interaction with the ligand binding domain of the androgen receptor. J Biol Chem. 2000;275:22986-22994. 10816582 10.1074/jbc.M002807200

[3] He B Bowen NT Minges JT Wilson EM. Androgen-induced NH2- and COOH-terminal Interaction Inhibits p160 coactivator recruitment by activation function 2. J Biol Chem. 2001;276:42293-42301. 11551963 10.1074/jbc.M107492200

[4] He B Gampe RT Kole AJ et al Structural basis for androgen receptor interdomain and coactivator interactions suggests a transition in nuclear receptor activation function dominance. Mol Cell. 2004;16:425-438. 15525515 10.1016/j.molcel.2004.09.036

[5] Zboray L Pluciennik A Curtis D et al Preventing the androgen receptor N/C interaction delays disease onset in a mouse model of SBMA. Cell Rep. 2015;13:2312-2323. 26673324 10.1016/j.celrep.2015.11.019 PMC4684905

[6] Langley E Kemppainen JA Wilson EM. Intermolecular NH2-/carboxyl-terminal interactions in androgen receptor dimerization revealed by mutations that cause androgen insensitivity. J Biol Chem. 1998;273:92-101. 9417052 10.1074/jbc.273.1.92

[7] van Royen ME Cunha SM Brink MC et al Compart-mentalization of androgen receptor protein-protein interactions in living cells. J Cell Biol. 2007;177:63–72. 17420290 10.1083/jcb.200609178 PMC2064112

[8] Li J Fu J Toumazou C Yoon HG Wong J. A role of the amino-terminal (N) and carboxyl-terminal (C) interaction in binding of androgen receptor to chromatin. Mol Endocrinol. 2006;20:776-785. 16373397 10.1210/me.2005-0298

[9] Tetel MJ Giangrande PH Leonhardt SA McDonnell DP Edwards DP. Hormone-dependent interaction between the amino- and carboxyl-terminal domains of progesterone receptor in vitro and in vivo. Mol Endocrinol. 1999;13:910-924. 10379890 10.1210/mend.13.6.0300

[10] Métivier R Petit FG Valotaire Y Pakdel F. Function of N-terminal transactivation domain of the estrogen receptor requires a potential alpha-helical structure and is negatively regulated by the A domain. Mol Endocrinol. 2000;14:1849-1871. 11075817 10.1210/mend.14.11.0546

[11] Rogerson FM Fuller PJ. Interdomain interactions in the mineralocorticoid receptor. Mol Cell Endocrinol. 2003;200:45-55. 12644298 10.1016/s0303-7207(02)00413-6

[12] Kobayashi K Sueyoshi T Inoue K Moore R Negishi M. Cytoplasmic accumulation of the nuclear receptor CAR by a tetratricopeptide repeat protein in HepG2 cells. Mol Pharmacol. 2003;64:1069-1075. 14573755 10.1124/mol.64.5.1069

[13] Ohno M Kanayama T Moore R Ray M Negishi M. The roles of co-chaperone CCRP/DNAJC7 in Cyp2b10 gene activation and steatosis development in mouse livers. PLoS ONE. 2014;9:e115663. 25542016 10.1371/journal.pone.0115663 PMC4277317

[14] Squires EJ Sueyoshi T Negishi M. Cytoplasmic localization of pregnane X receptor and ligand-dependent nuclear translocation in mouse liver. J Biol Chem. 2004;279:49307-49314. 15347657 10.1074/jbc.M407281200

[15] Moffatt NS Bruinsma E Uhl C Obermann WM Toft D. Role of the cochaperone Tpr2 in Hsp90 chaperoning. Biochemistry. 2008;47:8203-8213. 18620420 10.1021/bi800770g

[16] Schülke JP Wochnik GM Lang-Rollin I et al Differential impact of tetratricopeptide repeat proteins on the steroid hormone receptors. PLoS ONE. 2010;5:e11717. 20661446 10.1371/journal.pone.0011717 PMC2908686

[17] Brychzy A Rein T Winklhofer KF Hartl FU Young JC Obermann WM. Cofactor Tpr2 combines two TPR domains and a J domain to regulate the Hsp70/Hsp90 chaperone system. EMBO J. 2003;22:3613-3623. 12853476 10.1093/emboj/cdg362 PMC165632

[18] Wittig I Braun HP Schägger H. Blue native PAGE. Nat Protoc. 2006;1:418-428. 17406264 10.1038/nprot.2006.62

[19] He B Wilson EM. Electrostatic modulation in steroid receptor recruitment of LXXLL and FXXLF motifs. Mol Cell Biol. 2003;23:2135-2150. 12612084 10.1128/MCB.23.6.2135-2150.2003 PMC149467

[20] Combet C Blanchet C Geourjon C Deléage G. NPS@: network protein sequence analysis. Trends Biochem Sci. 2000;25:147-150. 10694887 10.1016/s0968-0004(99)01540-6

[21] Spanjaard RA Chin WW. Reconstitution of ligand-mediated glucocorticoid receptor activity by *trans*-acting functional domains. Mol Endocrinol. 1993;7:12-16. 8446102 10.1210/mend.7.1.8446102

[22] Johnson JL Craig EA. A role for the HSP40 Ydj1 in repression of basal steroid receptor activity in yeast. Mol Cell Biol. 2000;20:3027-3036. 10757787 10.1128/mcb.20.9.3027-3036.2000 PMC85575

[23] Hedman E Widén C Asadi A et al Proteomic identification of glucocorticoid receptor interacting proteins. Proteomics. 2006;6:3114-3126. 16619302 10.1002/pmic.200500266

[24] Dewint P Gossye V De Bosscher K et al A plant-derived ligand favoring monomeric glucocorticoid receptor conformation with impaired transactivation potential attenuates collagen-induced arthritis. J Immunol. 2008;180:2608-2615. 18250472 10.4049/jimmunol.180.4.2608

