# Supplementary figures and images for: GR Utilizes a Co-Chaperone Cytoplasmic CAR Retention Protein to Form an N/C Interaction
Source: Nucl Recept Signal. 2018 Oct 24;15:1550762918801072. doi: 10.1177/1550762918801072 (PMC6348740; doi:10.1177/1550762918801072)

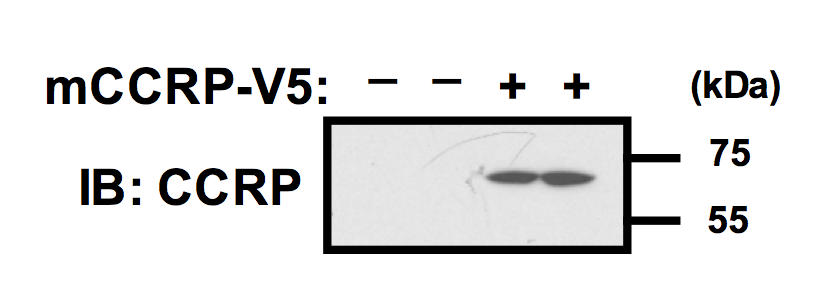

Supplement: Ohno_Supplemental_Fig_1 – Supplemental material for GR Utilizes a Co-Chaperone Cytoplasmic CAR Retention Protein to Form an N/C Interaction [file Ohno_Supplemental_Fig_1.tiff]

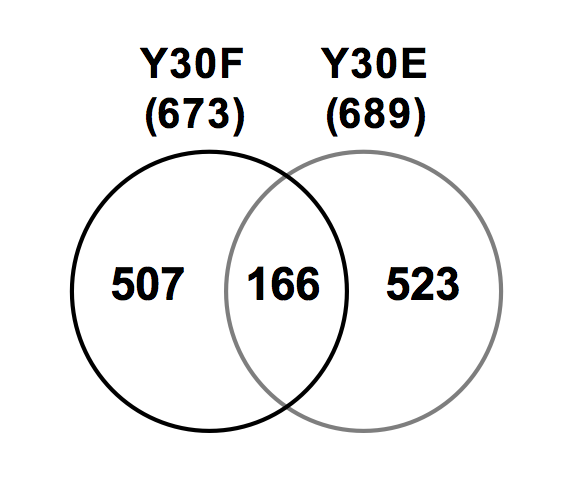

Supplement: Ohno_Supplemental_Fig_2 – Supplemental material for GR Utilizes a Co-Chaperone Cytoplasmic CAR Retention Protein to Form an N/C Interaction [file Ohno_Supplemental_Fig_2.tiff]
