# Supplementary material for: GR Utilizes a Co-Chaperone Cytoplasmic CAR Retention Protein to Form an N/C Interaction
Source: Nucl Recept Signal. 2018 Oct 24;15:1550762918801072. doi: 10.1177/1550762918801072 (PMC6348740; doi:10.1177/1550762918801072)
Supplement: Supplemental_Figure_Captions – Supplemental material for GR Utilizes a Co-Chaperone Cytoplasmic CAR Retention Protein to Form an N/C Interaction [file Supplemental_Figure_Captions.pdf]

Supplemental Figure Captions

Supplemental figure 1. Endogenous CCRP is undetectable in COS-1 cells.

Western blot analysis showed levels of the endogenous CCRP and of the ectopically expressed CCRP-V5 in COS-1 cells. Forty hours post transfection with pcDNA3.1-V5 or CCRP-V5 expression vector, total lysates were prepared. Fifteen micrograms of protein were applied to each lane, followed by Western blot analysis. Anti-CCRP antiserum was used to detect both endogenous CCRP and ectopically expressed CCRP-V5 as previously reported (Kobayashi et al., 2003; Ohno et al., 2014).

Supplemental figure 2. cDNA microarray analysis indicated that the GR N/C interaction may serve different gene regulation.

CCRP-V5 and FLAG-GR bearing either Y30F or Y30E mutation were transiently expressed in COS-1 cells, and cells were treated with vehicle (0.1% DMSO) or 100 nM dexamethasone for 6 hours at 37 °C in a 5% CO_2 incubator. Total RNAs were extracted from COS-1 cells using Trizol (Invitrogen) and purified with QIAGEN RNeasy kit (QIAGEN, Hilden, Germany) according to the manufacturer's protocols. Gene expression analysis was conducted using Agilent Whole Human Genome 4x44 multiplex format oligo arrays (Agilent Technologies, Santa Clara, CA) following the Agilent 1-color microarray-based gene expression analysis protocol as reported previously (Ohno et al., 2014). In order to identify differentially expressed probes, analysis of variance (ANOVA) was used to determine if there was a statistical difference between the means of groups. Gene tags were identified as dexamethasone responsive genes if fold change was greater than 1.5 and *p*-value was smaller than 0.05. The Venn diagram showed the number of genes which were significantly altered in

response to dexamethasone. GR Y30F and Y30E altered 673 and 689 genes, respectively in response to dexamethasone. Among them, only 166 genes were commonly regulated. It means that over 75% genes were uniquely regulated by either GR forms.
